# Case Report: Treatment of porphyria cutanea tarda with low dose hydroxychloroquine

**DOI:** 10.12688/f1000research.124022.1

**Published:** 2022-08-17

**Authors:** Andrew Awad, Alexander Nirenberg, Rodney Sinclair

**Affiliations:** 1 Sinclair Dermatology, East Melbourne, VIC, 3002, Australia; 2 Dorevitch Pathology, Heidelberg, VIC, 3084, Australia; 3 University of Melbourne, Parkville, VIC, 3010, Australia; 4 Epworth Healthcare, Richmond, VIC, 3121, Australia

**Keywords:** Porphyria cutanea tarda, hemochromatosis, hydroxychloroquine

## Abstract

**Background:** Porphyria cutanea tarda (PCT) is a complex metabolic disease resulting from altered activity of the enzyme uroporphyrinogen decarboxylase (UROD) in the liver resulting in accumulation of uroporphyrin. PCT presents as a blistering photodermatitis with skin fragility, vesicles, scarring and milia.

**Case:** We report a case of PCT in a 67-year-old man with hemochromatosis (HFE) gene mutation who, following a major syncopal episode in response to venesection was commenced on low dose hydroxychloroquine.

**Conclusions: **Low dose hydroxychloroquine provided a safe and effective alternative to venesection in this patient who was needle phobic.

## Introduction

Porphyria cutanea tarda (PCT) is the most common type of porphyria and results from a decline of uroporphyrinogen decarboxylase (UROD), an enzyme involved in heme synthesis and resulting in accumulation of uroporphyrin. ^1^ There are two types of PCT. Type 1 is an acquired disorder triggered by iron overload or viral infection. Type II is an autosomal-dominant genodermatosis. ^2^ The cutaneous manifestations arise when cutaneous porphyrins absorb ultraviolet radiation (UV) and generate reactive oxygen species (ROS) that induce skin photosensitivity, sub-epidermal blistering, erosions, milia and scar formation. ^3^ We present a case of a patient diagnosed with type 1 PCT and hemochromatosis, successfully treated with low dose hydroxychloroquine.

## Case report

**Figure 1.**
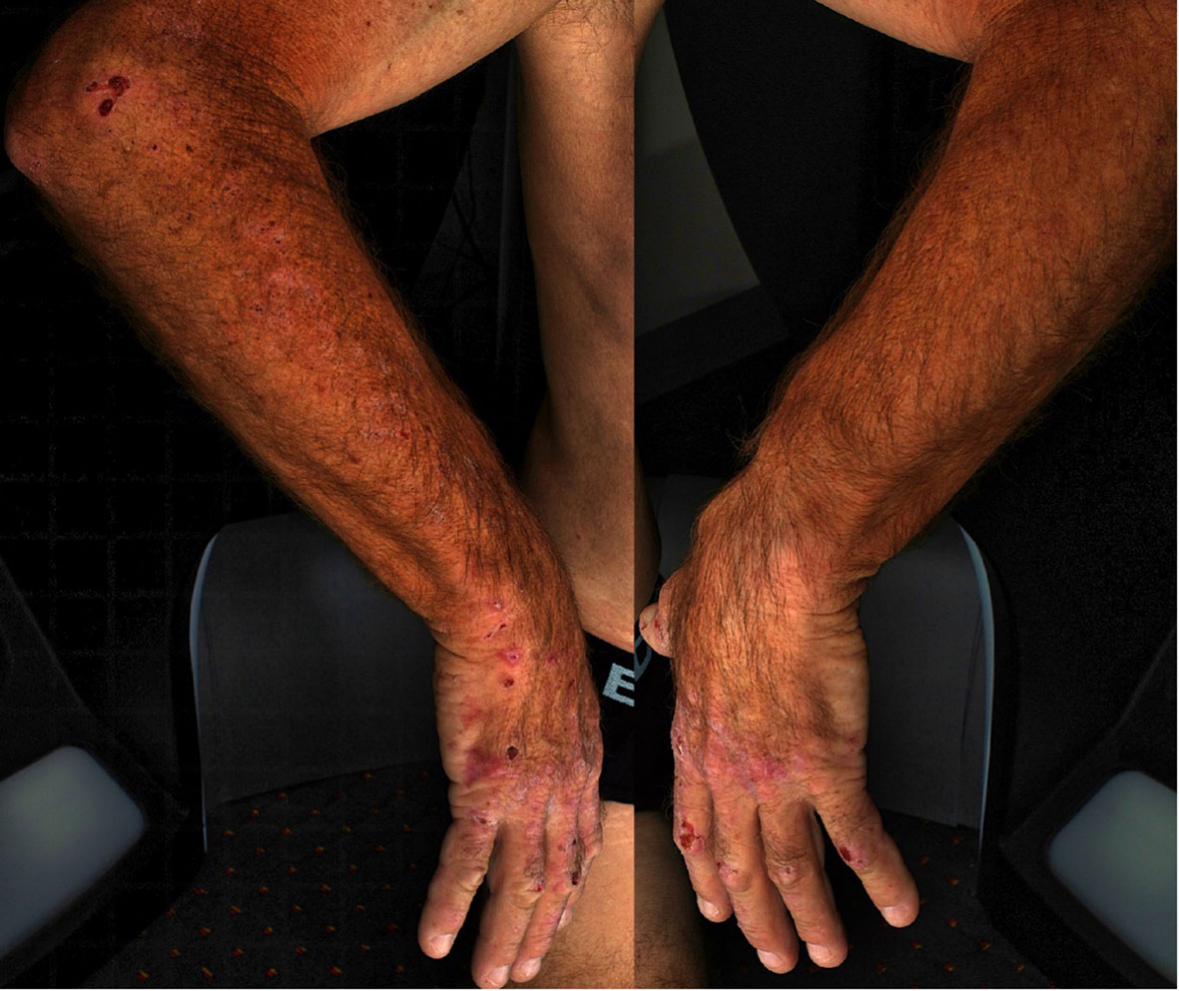
Appearance of the hands showing extensive erosions and intact blisters.

A 67-year-old Caucasian male, who worked as a plumber, presented with a two-month history of skin fragility and pruritic blisters on dorsal hands, spreading up the arms. He noted that broken blisters healed with firm little “white spots” and described lethargy and darkening of urine. For many years he had regularly consumed three cans of beer daily. Examination revealed intact blisters alongside numerous erosions over the hands and elbows and milia on inspection of the left hand ( Figure 1). There was no forehead hypertrichosis.

The clinical differential diagnosis included hepatocutaneous porphyria and epidermolysis bullosa acquisita (EBA). Skin biopsy, blood, urinary and faecal porphyrins were performed and he was advised to reduce alcohol consumption and sun exposure.

**Figure 2.**
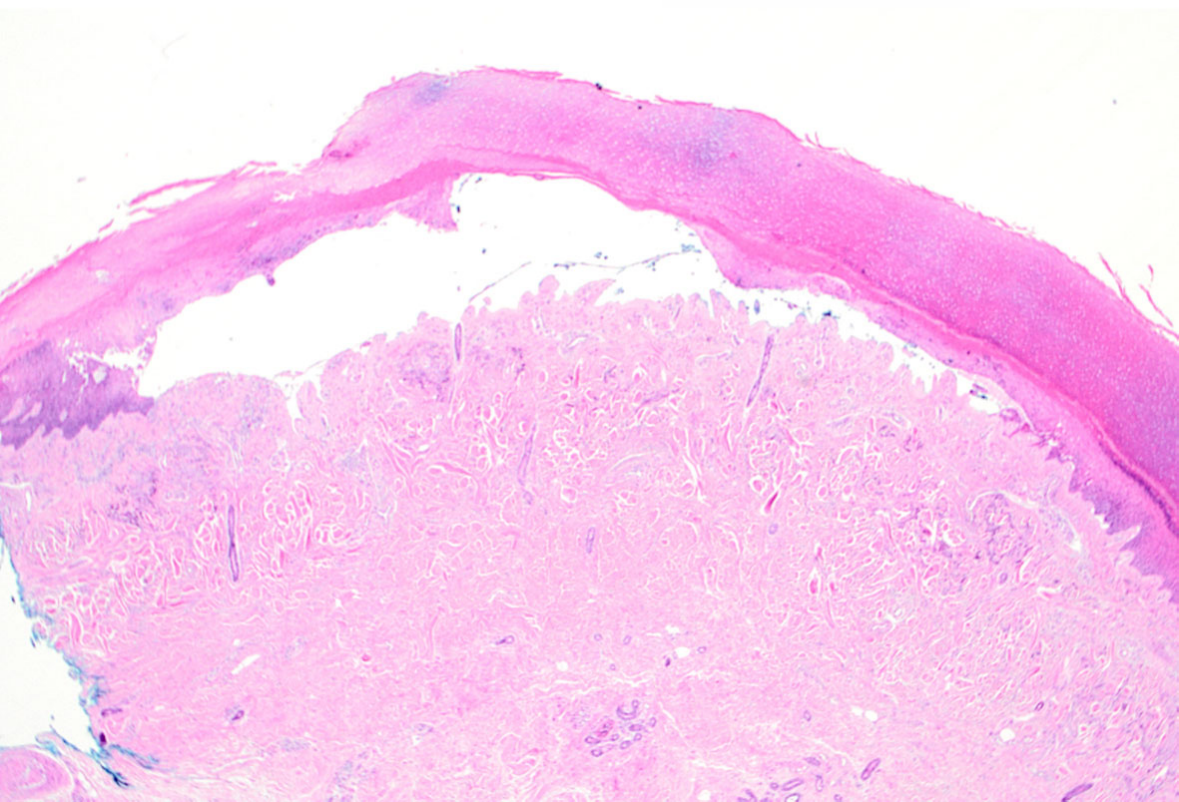
Punch biopsy of right hand demonstrating a sub-epidermal blister with festooning of dermal papilla and with adjacent epidermal hyperplasia with hypergranulosis. In the superficial dermis there are some thick-walled blood vessels, which stain for Periodic acid–Schiff (PAS). In the upper dermis there is a sparse lymphocytic infiltrate.

Histopathology revealed an intact sub-epidermal blister with minimal dermal inflammation ( Figure 2). Haematological investigations demonstrated; C282Y hemochromatosis (HFE) gene mutation, mildly elevated liver function tests (LFTs) consistent with alcohol intake and elevated ferritin (756 μg/L). Hepatitis and HIV serology were negative. The porphyrin screen showed elevated urinary total porphyrin (3.4 μmol/L), uroporphyrin (3.2 μmol/L), faecal total porphyrin (330 μmol/L), Isocoprophyrin (125 μmol/L), plasma porphyrin (207 nmol/L) and red cell porphyrin (1.8 μmol/L rbc). The patient was referred for venesection but had a major syncopal episode and declined further venesection. Treatment was then commenced with oral hydroxychloroquine 5 mg daily (compounded extemporaneously) and up-titrated over six months to 200 mg daily. Within 12 months the PCT was in complete remission with normal LFTs and ferritin (462 μg/L) and hydroxychloroquine was ceased. The porphyrin screen showed normal levels of urinary total porphyrin (0.17 μmol/L), red cell porphyrin (0.41 μmol/L red cells) and plasma total porphyrin (13 nmol/L).

## Discussion

The most common triggers for type 1 PCT are alcohol, environmental chemicals, hemochromatosis and viruses. ^2^ Our patient possessed two of the above triggering factors (alcohol and hemochromatosis). Alcohol may trigger PCT by increasing iron absorption through dissociating iron from its binding proteins as well as directly inhibiting UROD. ^4^ Hemochromatosis causes iron excess and can trigger PCT by catalysing ROS formation and increasing uroporphyrin concentration through direct oxidation of uroporphyrinogen and inhibition of UROD. ^1^

Hydroxychloroquine increases porphyrin excretion in PCT and is as effective as phlebotomy with remission in 6–9 months. ^5^ The HFE mutation type appears to be important in hydroxychloroquine therapeutic response, with homozygosity for the C282Y mutation resulting in treatment failure, but heterozygosity, as demonstrated in our case, did not. ^6^ The use of compounded low-dose hydroxychloroquine minimised risk of acute toxicity and provided a safe and effective alternative to venesection in our patient. The dose was up-titrated until clinical remission was achieved. A limitation of this case report was the small sample size of only one patient.

## Conclusions

Oral hydroxychloroquine provided a safe and effective alternative to venesection in this patient who was needle phobic.

## Data availability

### Underlying data

All data underlying the results are available as part of the article and no additional source data are required.

## Consent

Written informed consent for publication of their clinical details and clinical images was obtained from the patient.
