# Supplementary material for: Case Report: Treatment of porphyria cutanea tarda with low dose hydroxychloroquine
Source: F1000Res. 2022 Aug 17;11:945. [Version 1] doi: 10.12688/f1000research.124022.1 (PMC10285345; doi:10.12688/f1000research.124022.1)
Supplement: Peer review history

# Peer review: Reviewer response for version 1

**DOI:** 10.5256/f1000research.136191.r172322

**Published:** 2023-06-08

**Authors:** Andrea Ricci

**Affiliations:** 1 Department of Medical and Surgical Science for Children and Adults, Universita degli Studi di Modena e Reggio Emilia, Modena, Emilia-Romagna, Italy

The authors report an interesting case report which reminds us that hydroxychloroquine is an effective treatment for PCT. This should be kept in mind for all those patients who cannot undergo phlebotomy (for instance, cirrhotic patients, or those affected by myelodysplastic disorders) even though the choice to use hydroxychloroquine should be always weighed against the risk of adverse events in patients who are already affected by a liver disease.

 I find this report clearly written and informative. However, a few points should be corrected/clarified:

- This patient carried a heterozygous C282Y variant in the human homeostatic iron regulator protein (HFE) gene, which should be mentioned as such  (not " *hemochromatosis*" gene, as there are several other genes - HJV, TFR2, etc. - which cause hemochromatosis). A C282Y heterozygous mutation is by no means sufficient to cause HFE-related hemochromatosis, which is mainly associated with a C282Y homozygous genotype. A compound heterozygous C282Y/H63D genotype may be regarded as a "risk genotype" for liver iron overload, but is not sufficient in itself to define "classic" HFE-related hemochromatosis. In sum, the authors should consider that the iron overload phenotype of their patient cannot be explained by his HFE genotype (I assume that the patient did not carry further pathogenic variants in this gene). Furthermore, they should report the patient's serum iron, transferrin, and transferrin saturation levels at baseline (both serum iron and transferrin saturation should be above the range of normality in a typical hemochromatosis patient, whereas serum transferrin should be normal/below range).
- Did the patient change his habits as to daily ethanol consumption? As the authors hint, this could be the main reason for his iron overload phenotype.
- Isocoproporphyrin is misspelled.
- The patient did not undergo a liver biopsy, which is reasonable enough a choice, provided the hepatopathy he was affected by is reasonably diagnosed as alcohol-related. This should be specified somewhere in the text. If the authors think that alcohol use alone does not fully explain this patient's liver disease, they should clarify why other causes of hepatopathy (i.e. NAFLD, autoimmune, etc.) have not been investigated.

Are enough details provided of any physical examination and diagnostic tests, treatment given and outcomes?

Partly

Is the case presented with sufficient detail to be useful for other practitioners?

Yes

Is sufficient discussion included of the importance of the findings and their relevance to future understanding of disease processes, diagnosis or treatment?

Partly

Is the background of the case’s history and progression described in sufficient detail?

Yes

Reviewer Expertise:

Rare liver diseases, iron overload disorders (hemochromatosis, ferroportin disease, hemoglobinopathies), porphyrias

I confirm that I have read this submission and believe that I have an appropriate level of expertise to confirm that it is of an acceptable scientific standard, however I have significant reservations, as outlined above.

# Peer review: Reviewer response for version 1

**DOI:** 10.5256/f1000research.136191.r172317

**Published:** 2023-05-23

**Authors:** Staffan Wahlin

**Affiliations:** 1 Department of Upper GI Diseases, Karolinska University, Stockholm, Sweden

This is a short report about a fairly standard PCT patient who was successfully treated to reach clinical and biochemical remission.

 I have two main concerns:

1. The title of the report does not really reflect the content; " *low dose*" is true only in comparison with other non-PCT indications. In PCT, the recommended dose of hydroxychloroquine is 100 mg twice weekly (for chloroquine, 125 mg twice weekly). The reason for keeping the dose low is mainly a significant risk of hepatotoxicity in higher doses. Toxic retinopathy is also an issue. This case was treated with 5 mg daily uptrited to 200 mg daily. In a PCT context, that should be regarded as "high dose". The patient did ok with normal LFTs during the treatment period.
2. Please don't say that the patient had hemochromatosis. He had a HFE gene variant on one allele, he was heterozygous. The moderately elevated serum ferritin was more likely a result of alcohol overconsumption (>20 beer cans per week). His main risk factor was alcohol. The HFE mutation may have made him more vulnerable.

**Some details:**

- In Introduction: " *Type 1 is an acquired disorder triggered by* **risk factors such as** *iron overload...*"
- Porphyrins absorb mainly visible light around 405 nm (could be expressed as **"**longer wavelength UVA and visible light around 405 nm"). Saying UV radiation is misleading as it suggests that protection with UV blocking creams may help. They don't.
- Units. Are umol/L standard in Australia? In Europe, nmol/L and, in the US, mcg/dL is standard/preferred. Check what is most appropriate for the journal.
- The last sentence about the limitation is unnecessary.

Are enough details provided of any physical examination and diagnostic tests, treatment given and outcomes?

Partly

Is the case presented with sufficient detail to be useful for other practitioners?

Partly

Is sufficient discussion included of the importance of the findings and their relevance to future understanding of disease processes, diagnosis or treatment?

No

Is the background of the case’s history and progression described in sufficient detail?

Yes

Reviewer Expertise:

Liver diseases. Porphyria.

I confirm that I have read this submission and believe that I have an appropriate level of expertise to confirm that it is of an acceptable scientific standard, however I have significant reservations, as outlined above.

# Peer review: Reviewer response for version 1

**DOI:** 10.5256/f1000research.136191.r156729

**Published:** 2022-12-16

**Authors:** Jordi To-Figueras

**Affiliations:** 1 Clinical Biochemistry Department, Hospital Clinic of Barcelona, Faculty of Medicine, University of Barcelona, Barcelona, Spain

This is an OK addition to the literature of PCT. 

1. The authors should however clarify the haemochromatosis status. In the case report, they should indicate if the patient is heterozygous for the C282Y mutation (hence C282Y/WT?) and add the H63D status (WT/WT?). According to reviews (i.e. Kowdley *et al. *(2019) ^1^), HFE status would be defined by a C282Y homozygous or C282Y/H63D compound heterozygous. Please clarify.
2. 'Case report' section: " *red cell porphyrin*" should read as "red cell protoporphyrin"; " *plasma porphyrin*" should read as "plasma total porphyrin".

The case the authors reported adds to the PCT literature. Even if venesection is considered a treatment of choice for PCT that has been empirically proven to reduce overproduction of porphyrins through reduction of hepatic iron depots, it is an invasive procedure. Needle phobia of the patient induced consideration of low-dose oral chloroquine as an alternative. And that simple and well-known regime induced remission even if haemochromatosis was suspected. The point is that, PCT being a benign disease, non-invasive treatments (chloroquine or oral antivirals when PCT is induced by HCV infection) should be preferred to venesection as a first choice.

Are enough details provided of any physical examination and diagnostic tests, treatment given and outcomes?

Partly

Is the case presented with sufficient detail to be useful for other practitioners?

Yes

Is sufficient discussion included of the importance of the findings and their relevance to future understanding of disease processes, diagnosis or treatment?

Yes

Is the background of the case’s history and progression described in sufficient detail?

Yes

Reviewer Expertise:

Porphyria

I confirm that I have read this submission and believe that I have an appropriate level of expertise to confirm that it is of an acceptable scientific standard.

## References

[1] : ACG Clinical Guideline: Hereditary Hemochromatosis. *Am J Gastroenterol* .2019;114(8) : 10.14309/ajg.0000000000000315 1202-1218 10.14309/ajg.0000000000000315 31335359
